# Integrated Features for Optimizing Machine Learning Classifiers of Pediatric and Young Adults With a Post-Traumatic Headache From Healthy Controls

**DOI:** 10.3389/fpain.2022.859881

**Published:** 2022-05-17

**Authors:** Scott Holmes, Joud Mar'i, Laura E. Simons, David Zurakowski, Alyssa Ann LeBel, Michael O'Brien, David Borsook

**Affiliations:** ^1^Pediatric Pain Pathway Lab, Department of Anesthesia, Critical Care, and Pain Medicine, Boston Children's Hospital – Harvard Medical School, Boston, MA, United States; ^2^Pain and Affective Neuroscience Center, Boston Children's Hospital, Boston, MA, United States; ^3^Department of Anesthesiology, Perioperative, and Pain Medicine, Stanford University School of Medicine, Palo Alto, CA, United States; ^4^Department of Anesthesia, Critical Care, and Pain Medicine, Boston Children's Hospital, Boston, MA, United States; ^5^Sports Medicine Division, Sports Concussion Clinic, Orthopedic Surgery, Harvard Medical School, Boston, MA, United States; ^6^Departments of Psychiatry ad Radiology, Massachusetts General Hospital and Harvard Medical School, Boston, MA, United States

**Keywords:** machine learning, MRI, post-traumatic headache, pain, pediatrics

## Abstract

Post-traumatic headache (PTH) is a challenging clinical condition to identify and treat as it integrates multiple subjectively defined symptoms with underlying physiological processes. The precise mechanisms underlying PTH are unclear, and it remains to be understood how to integrate the patient experience with underlying biology when attempting to classify persons with PTH, particularly in the pediatric setting where patient self-report may be highly variable. The objective of this investigation was to evaluate the use of different machine learning (ML) classifiers to differentiate pediatric and young adult subjects with PTH from healthy controls using behavioral data from self-report questionnaires that reflect concussion symptoms, mental health, pain experience of the participants, and structural brain imaging from cortical and sub-cortical locations. Behavioral data, alongside brain imaging, survived data reduction methods and both contributed toward final models. Behavioral data that contributed towards the final model included both the child and parent perspective of the pain-experience. Brain imaging features produced two unique clusters that reflect regions that were previously found in mild traumatic brain injury (mTBI) and PTH. Affinity-based propagation analysis demonstrated that behavioral data remained independent relative to neuroimaging data that suggest there is a role for both behavioral and brain imaging data when attempting to classify children with PTH.

## Introduction

Post-traumatic headache (PTH) is a neurological condition that impacts a large percentage of persons after a mild traumatic brain injury (mTBI) and personifies the opaque nature of pain symptom reporting. Persons with PTH can experience different headache characteristics that include tension-type headache, occipital neuralgia, cluster headache, and migraine (1). Of those who suffer an mTBI, approximately 15% continue to experience PTH at 3 months post-injury (2). Headache associated with PTH is usually accompanied by other symptoms, such as depression and anxiety (3) and pain (4), which can lead to considerable disability. The clinical reqchanges in the grey and white matter that typically resolve around tuirements for PTH necessitate that the headache is present for more than 7 days post-injury (3), suggesting a lingering element that can have a dynamic presence – being exacerbated by stress (5). PTH represents a challenging symptom to manage as it is reliant on accurate patient symptom reporting. With the development of more objective measures that include machine learning (ML) classifiers, it remains unclear how pediatric self-reports should be applied in the clinical setting.

Post-traumatic headache is associated with a breadth of behavioral symptoms and has been correlated with underlying neurostructural and neurofunctional alterations. Pediatric subjects with PTH often present with symptoms that can span the categories of somatic, vestibular, emotional, cognitive, and sleep symptoms (6). In the acute setting there may be variable changes in the grey or white matter of persons with PTH (7); however, in the chronic setting (>3 months) there are documented changes in the grey and white matter that typically resolve around the 1-year mark post-injury (8). There is evidence of correlations between headache frequency and cortical thickness (8, 9) and that certain neurofunctional features, such as the functional connectivity of the periaqueductal gray and precuneus (10) and the interaction between the salience and memory networks (11), are implicated as biomechanisms of symptom progression. Interesting work has been done by Schwedt et al. to understand the diversity of neurostructural and neurofunctional structures that are implicated in PTH [see (12)]. There has been one previous investigation that evaluates PTH relative to Migraine participants with ML, showing that behavioral markers of depression and concussion severity were integral in classifying PTH participants alongside characteristics of white matter pathways relative to persons with migraine (13). To date, there has been no research done on differentiating pediatric subjects with PTH from healthy controls.

We do not currently understand the mechanisms underlying PTH. The use of ML classifiers in mTBI has been previously explored using clinically oriented (14, 15) and neuroimaging (16, 17) data with variable success rates (accuracy range 72–94%). In adults with PTH, Schwedt et al. have shown that ML can, on average, be improved by including both subjective and objective data when comparing participants with a PTH to a migraine cohort (13). Children as young as 5 have been able to accurately report health-related quality of life (18), suggesting their perspective should be integrated into classification tools. It remains to be determined how pediatric self-reporting should be integrated into classification models attempting to delineate persons with a neurological injury from healthy controls. In the following investigation we evaluated (1) how subjective and objective data contributed to classify persons with PTH relative to healthy controls, (2) the role of different types of classifiers in predicting persons with PTH relative to healthy controls, and (3) how a reduced set of features cluster to classify pediatric and young adult subjects with PTH relative to healthy controls.

## Methods

### Subjects

**Figure 1:**
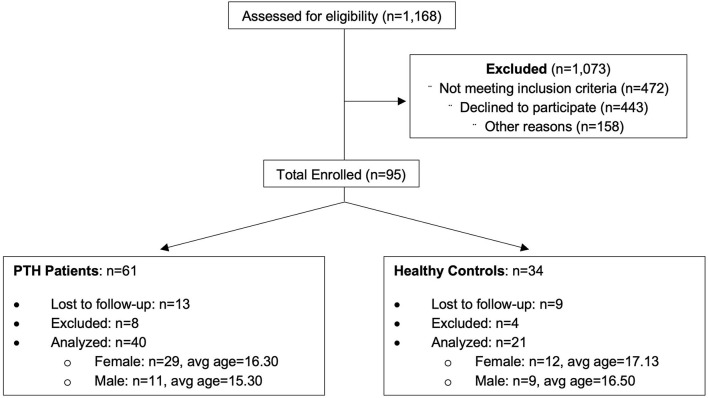
Consort diagram showing included/excluded participants in this investigation.

Participants were recruited from the greater Boston area as part of a 5-year study evaluating PTH in youth. An overview of recruitment can be found in Figure 1 and Table 1. Eligible participants were identified through a Power Chart review of all patients presenting to the Boston Children's Hospital Sports Medicine Clinic at Boston and Waltham, MA, USA or self-referral *via* community advertisements and flyers hung around college campuses in the Longwood Medical Area and on Partners Health Care portal. All patients with PTH fulfilled the International Classification of Diseases, Ninth Revision criteria for mTBI, completed a neurological examination (confirmed by neurologist), and reported to have developed a headache within 7 days after mTBI. Patients who had their headache symptoms conclude within 2 weeks to 1 month after the injury and no longer experienced PTHs and after the 1-month mark were placed in the resolved group. Headache recovery was determined through patient self-reporting. Persons in the Persistent cohort were still experiencing post-traumatic headaches past the 1-month mark post injury. This division was performed to clearly distinguish persons with resolved vs. persistent symptoms, although the official designation of persistence was maintained at 3 months from point of injury. The resolved and persistent groups were combined as one group of patients with mTBI and compared to healthy controls. This was performed to account for persistent abnormalities in brain structure in asymptomatic persons previously found in this cohort (11) and others (19). A total of 61 age- and sex-matched subjects (mTBI patients *n* = 40, 29 women; and healthy control subjects *n* = 21, 12 women) were analyzed and all underwent imaging at Boston Children's Hospital in Waltham, MA, USA. All subjects were right-handed and between ages 12 and 24 at the time of participation with no significant history of pre-existing headaches, chronic pain, or psychiatric neurological conditions (such as clinical depression or anxiety). Enrolled participants were screened for drugs of abuse (such as barbiturates, benzodiazepines, amphetamines, and tetrahydrocannabinol) and medications that would interfere with study findings. The study protocol was approved by the Institutional Review Board at Boston Children's Hospital and conducted in accordance with the principles of the Declaration of Helsinki. Informed consent and assent were obtained from all subjects prior to enrollment.

**Table 1:** Participant demographics.

	**PTH**	**Control**
Total N	40	21
Female N	29	12
Avg Age (years) [age range]	16.03 (2.56) [12.02–21.88]	16.86 (2.66) [12.64–22]
Avg days Since Injury	89.9 (28.41)	NA
Avg Impact Total Score	11.39 (17.58)	1.4 (2.06)
Avg Impact Headache Score	1.12 (1.5)	0

*Means and standard deviations are provided for participant demographics and psychological testing at the first study visit*.

### Psychological Questionnaires and Testing

At the time of each study visit, each participant met with a physician or registered nurse to review eligibility and concussion symptomology. The following questionnaires were completed by both mTBI patients and healthy controls cohorts: depression (Childhood Depression Inventory [CDI]; age <18), Beck Depression Inventory (BDI; age >18), Revised Children's Manifest Anxiety Scale (RCMAS), Pubertal Developmental Scale (PDS), Pain Catastrophizing Scale (PCS), Childhood Anxiety Sensitivity Index (CASI), Child and Parent Fear of Pain Questionnaire (FOPQ) filled by patients and their parents, Pediatric Pain Screening Tool (PPST), and Immediate Post-concussion Assessment and Cognitive Testing (IMPACT) concussion survey. T-scores were extracted and used from the CDI and BDI and integrated into the Depression metric. T-scores for the BDI were obtained using scoring for the Patient Reported Outcomes Measurement Information System depression subscale. mTBI patient cohort received two additional questionnaires: the Rivermead Concussion Survey and the Allodynia Symptom Checklist (ASC-12) to assess for post-concussive symptoms and sensitivity to painful stimuli respectively.

### Magnetic Resonance Imaging (MRI) Data Acquisition

Magnetic resonance imaging data were collected on a 3T Siemens MAGNETOM Trio Tim scanner with a 12-channel phased-array head coil (Erlangen, Germany). T1 Magnetization-Prepared Rapid Acquisition Gradient-Echo (MPRAGE) anatomical images were collected using a gradient echo-echo planar pulse sequence with 1.0 × 1.0 × 1.0 mm resolution. MPRAGE scan parameters consisted of the following: repetition time (TR) = 2,520 msec; echo time (TE) = 1.74, 3.54, 5.34, 7.14 msec; field of view (FOV) = 220· × 220; flip angle (FA) = 7°; and axial slices = 176. Resting-state functional connectivity (RS-FC) data were collected using a gradient echo-echo planar pulse sequence with 3.0 × 3.0 × 3.0 mm resolution. Functional MRI (fMRI) scan parameters consisted of the following: TR = 1,100 msec; TE = 30 msec; FOV = 228 × 228; FA = 70°; axial slices = 51; volumes = 320; and acquisition time = 6 min. Patients were instructed to remain still, clear their minds, and keep their eyes open during the scan sequence.

### Structural Brain Data

Cortical reconstruction and volumetric segmentation were performed with FreeSurfer (version 5.3) image analysis suite. Once the cortical models were completed, parcellation of the cerebral cortex into regional units was finished. This method produced representations of cortical thickness and subcortical volume extracted from T1-weighted images. For data analyzed in this study, all surfaces were visually checked, and manual interventions were used as needed to correct small defects.

### Data Gathering and Preparation

The full data set used as the initial unprocessed input for this ML investigation included raw brain imaging and psychological data combined. For the brain imaging data, cortical and subcortical volume measures were used with a total of 70 extracted regions of interest (ROIs). For psychological data, measures related to pain levels (PCS, FOPQ-C, FOPQ-P, and PPST) and head injury (IMPACT total scored and IMPACT headache subscale) were used. The combined brain and psychological data also collapsed across groups (mTBI patients and healthy controls together) and study visits (all 4 time points together). Thus, the full data set consisted of columns that represent the 70 ROIs and 6 psychological measures and rows that represent each subject across groups and time points. Variables with missing values were eliminated reducing the number of individual timepoints from 239 to 228. Feature scaling was conducted by standardizing and converting all values to Z-scores. Python 3.9 was used to convert raw values to Z-scores and produces the initial Data Frame to be used for the rest of the investigation. For all the following analyses explained below, Python 3.9 was used with Jupyter Notebook code scripts provided on github (https://github.com/scottneuro/ML_PTH_Headache/upload). The libraries used in this article include NumPy, pandas, Matplotlib, Seaborn, SciPy, and the ML Scikit-learn library.

### Data Reduction

Pearson correlation from the Pandas library was used to compute the pairwise correlation of columns to each other (ROIs and psychological measures). A correlation matrix of the absolute values of standard correlation coefficients was then created, where columns with correlation coefficients >0.5 were dropped. Higher correlations meant that these variables/columns were too like another and thus can be dropped. This helps in avoiding redundancy and model overfitting by improving the robustness of training and testing ML classifiers and allowing them to generalize well on unseen data. A resulting reduced Data Frame was created with the left selected features.

### Data Splitting

In the reduced data set, the target column was set to Group. The goal was to assess the ability of various models to generalize on unseen data by accurately predicting in which group (mTBI or patient) a new subject would fall under from their new subcortical volume and psychological data. To do so, the data set was first split into two, training and testing sets, using the Scikit learn library train test split tool. The function splits the reduced data set matrix into random train and test subsets. The training set used 80% of the reduced data to train the following models presented below. The testing set used the rest of the 20% of the reduced data to test the accuracy of the models' predictions.

### Data Modeling and Analytics

After the training and testing subsets were ready, they were fitted into the following models: support vector machines (SVMs; with a linear kernel), k-nearest neighbor (KNN; K = 5), and decision trees (DTs). These three models were used from the Scikit learn library of classifiers in Python. Model predictions were done using the test data subset only. The accuracy score of each model was calculated using the ratio of true positives and true negatives from all predictions. Receiver Operating Characteristic (ROC) curves were generated for each model to show the predictive accuracy of each of these binary classifiers.

### Feature Selection and Clustering

After performing data reduction using Pearson correlation and training/testing the reduced data using the 3 different classifiers, we wanted to know the specific contribution of each of these reduced variables to the target and which ROI or psychological measures best predict the group a new subject would be put into. To do this, we used the Univariate Selection, SelectKBest, method, where the features with the strongest relationships to the target output variable are selected. SelectKBest class selects a specific number of features and uses the chi-squared (chi^2^) test for non-negative features to select x number of the best features from the data. After performing feature selection, we were also interested in exploring the relationship between these reduced salient features to see what ROIs/psychological data would cluster together. Hierarchical Clustering using SciPy's Agglomerative Clustering method was used to generate corresponding dendrograms.

## Results

### Participant Characteristics

A list of participant demographics and injury characteristics is provided in Table 1. Participants included persons with an mTBI resulting from sports, falls, and accidents. Brain imaging and questionnaire data were included in the classification analysis from 238 individual time points. A total of 158 individual time points were evaluated for all participants with PTH and 80 individual time points were provided for healthy controls and were divided as follows: 3-month (*n* = 64), 6-month (*n* = 62), 9-month (*n* = 62), and 12-month (*n* = 50) data.

### Data Reduction Heatmaps

**Figure 2:**
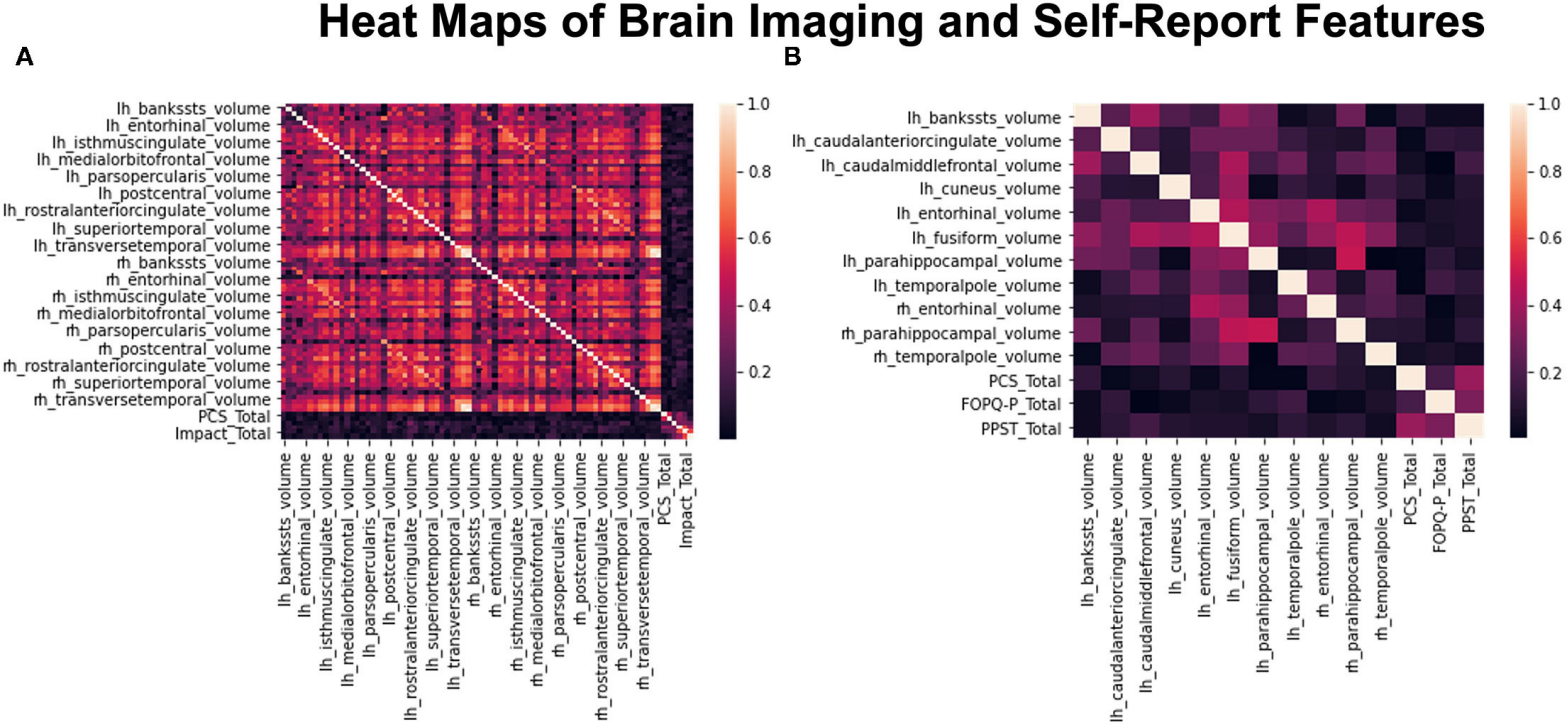
Heat maps before and after data reduction. **(A)** Shows the compiled heatmap of all 76 features before dropping out the highly correlated variables. **(B)** Shows the heatmap of reduced dataset with only 14 features left after dropping highly correlated variables.

Pearson correlation was successfully able to reduce the original initial input dataset that contains 70 ROIs and 6 psychological metrics into 14 salient features (Figure 2). From these 14 reduced features, which are used in the rest of the analysis steps, there are 11 ROIs and 3 psychological measures as shown in Figure 2. The brain regions were superior temporal sulcus – left hemisphere, caudal anterior cingulate – left hemisphere, caudal middle frontal – left hemisphere, cuneus – left hemisphere, entorhinal – left and right hemispheres, fusiform – left hemisphere, parahippocampal – left and right hemispheres, temporal pole – left and right hemispheres, PCS, FOPQ-P, and PPST.

### Prediction Accuracy of Models

**Figure 3:**
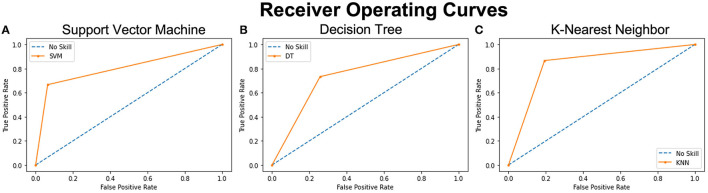
ROC curves of 3 analyzed models representing the prediction accuracy of each. The orange curves show the trade-off between sensitivity (True Positive Rate – TPR on y axis) and specificity (False Positive Rate – FPR on x axis). Curves closer to the left-top corner indicate better performance. The blue dashed lines – No Skill, are the baseline diagonals (FPR = TPR), where classifiers are expected to give point lying on this line. The closer the orange curves are to the 45 degrees blue diagonal, the less accurate the model is. **(A)** Shows the ROC curve of the SVM model with the highest accuracy score and the closest tip to the y-axis and the left-top corner. **(B)** shows the ROC curve of the Decision Tree analysis with the lowest accuracy score and **(C)** shows the ROC curve of the KNN model with the second highest accuracy score.

Across the three models used to train and test the reduced dataset of 14 selected features, SVM had the highest prediction accuracy of classifying persons with PTH from healthy controls with a score of 0.85. KNN came second with a score of 0.83 and DTs came last with a score of 0.74. ROC curves outlined in Figure 3 highlight similar trends between the three ML models. The SVM model provided a slightly better positivity rate for false positives; however, the KNN and deep learning (DL) models performed with similar true- and false-positive rates.

### Feature Contribution to the Models

Using the SelectKBest Univariate Feature Selection method (Table 2), FOPQ-P psychological measure had the highest score among all 14 features. It has the most contribution and best ability to predict the group of a new subject when compared to the rest of the 13 features. The superior temporal sulcus had the lowest score and contribution. Notably, a reduction of the classifiers to include only those with factor contributions >1 did not improve the model accuracy.

**Table 2:** Importance or Contribution scores of the 14 reduced selected features by the SelectKBest feature selection method.

**Feature**	**Score**
FOPQ-P	4.663250
LH Caudal Middle Frontal	3.702541
LH Cuneus	2.668843
RH Entorhinal	2.551437
LH Fusiform	1.233778
PPST	1.178420
LH Para Hippocampal	0.398127
PCS	0.354847
RH Para Hippocampal	0.139298
LH Temporal Pole	0.094458
LH Caudal Anterior Cingulate	0.070233
RH Temporal Pole	0.022997
LH Entorhinal	0.020928
LH Superior Temporal Sulcus	0.002227

### Hierarchical Clustering

**Figure 4:**
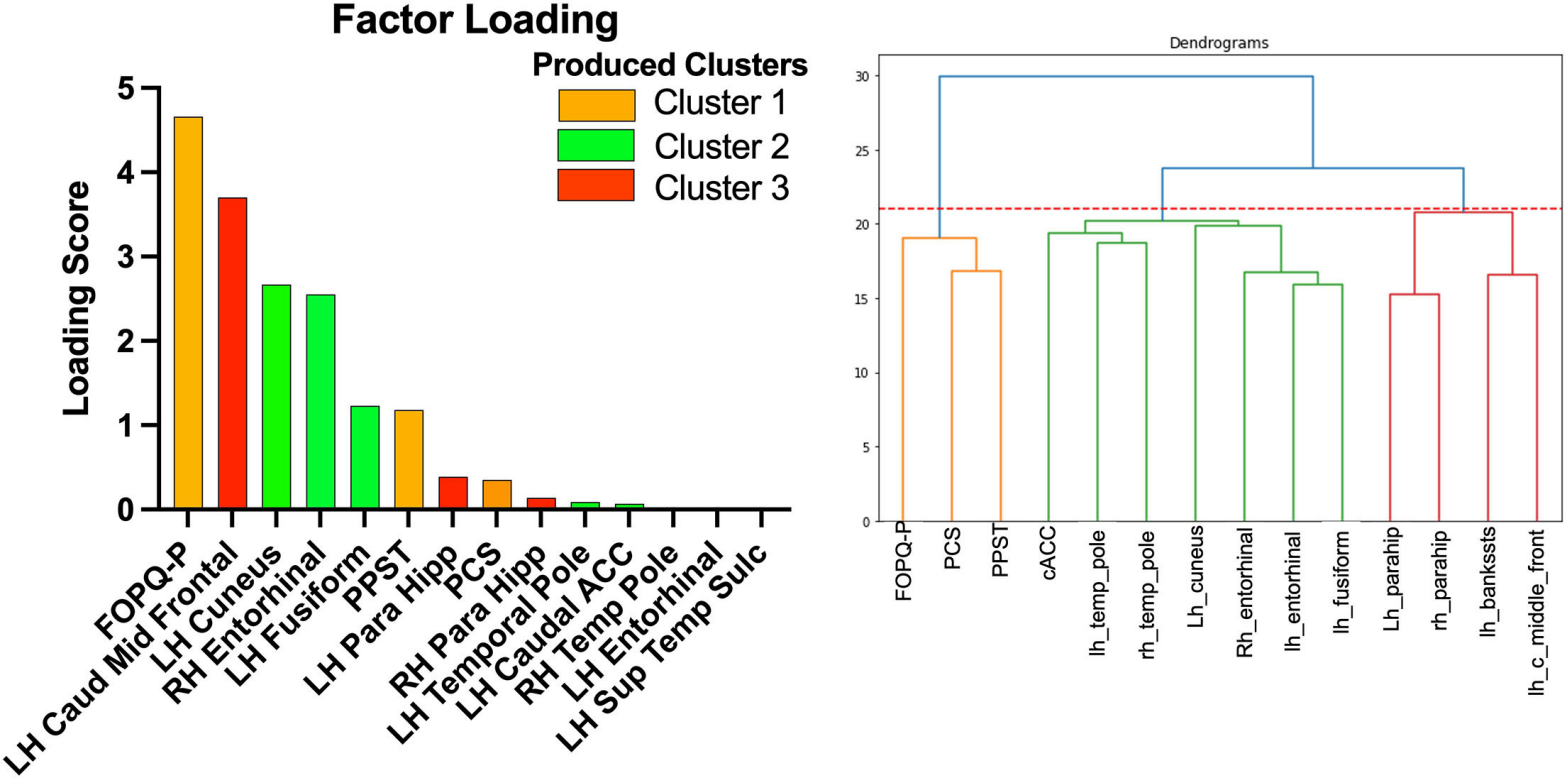
Factor loading (left) and dendrogram (right) showing the clustering of the 14 selected variables. The orange cluster is the psychological data. The green cluster is cluster 2 and the red cluster is cluster 1.

The Agglomerative Clustering method was performed to evaluate how each of the 14 selected features cluster (Figure 4). Psychological data were all grouped together in one cluster that includes the 3 features (PPST, FOPQ-P, and PCS). Brain data were split into 2 clusters. Cluster 1 included the superior temporal sulcus – left hemisphere, caudal middle frontal – left hemisphere, and parahippocampal – left and right hemisphere. Cluster 2 was composed of the remaining brain regions that included the caudal anterior cingulate – left hemisphere, cuneus – left hemisphere, entorhinal – left and right hemispheres, fusiform – left hemisphere, and temporal pole – left and right hemispheres.

## Discussion

Headache biomechanisms in pediatrics are poorly understood. It remains unclear how objective and subjective data contribute toward understanding PTH in pediatric and young adult subjects who have suffered an mTBI. The pathology underlying mTBI impacts multiple cortical regions (20), and has been shown in PTH as well (12). The question of how to incorporate their self-report symptoms has not yet been addressed. Accurate reporting is dependent on a subjective rating and has been shown reliable in pediatrics (21); however, it is unclear how self-report contributes toward patient classification. Findings from this investigation (1) emphasize pediatric and young adult self-reporting and caregiver reporting, (2) highlight unique brain clusters that may underscore mTBI vs. PTH mechanisms, and (3) how subjective and objective features cluster in unique patterns.

Pain-focused metrics contribute to classify pediatric and young adult subjects with PTH from healthy controls. The presence and extent of PTH are currently subjectively defined through patient self-reporting (3) and parallel that of pain (22) as there is no definitive objective test. The behavioral symptoms that can accompany persons with PTH can be diverse, such as somatic, vestibular, emotional, cognitive, and sleep-related, with a potential sex-based effect (6), and persons as low as 5 years of age have been shown to provide reliably self-reporting of quality of life (18). The use of the PPST, a screening tool for the identification of pain in youth (23), highlights that pain is a prominent factor in youth with PTH [see also (11)]. The PCS was developed to understand the presence of an “exaggerated negative mental set” (24), further underscoring the vulnerable mental health state of youth. Interestingly, the inclusion of Fear of Pain reporting from parental/caregiver sources in the final model highlights a role for how caregivers are involved in the mTBI and PTH diagnostic process, perhaps alluding to parents/caregivers providing exaggerated reportings (25) or perhaps demonstrating a role for experienced advocacy. The absence of features that reflect the extent of mTBI symptoms or mental health concerns, such as anxiety or depression, is likely the result of headache-focused recruitment and redundant feature elimination and the use of screening for mental health prior to study enrollment. As such, we show a strong role for the use of pain screening in youth with suspected PTH.

Brain regions contribute to the models align with both the mTBI and PTH literature. There was a total of 11 brain regions that contributed toward the optimal classification of patients from healthy controls. The structural neuroimaging features are grouped into two clusters. In one cluster, we observed the caudal middle frontal and parahippocampal regions from the left and right hemispheres with the superior temporal sulcus. The caudal middle frontal gyrus has previously been found in persons with PTH (26) and may represent the visual disturbances in persons with mTBI and headache that can include photosensitivity and photophobia (27). The superior temporal sulcus is a region associated with multisensory integration, such as touch sound and vision (28, 29), all symptoms impacted commonly in mTBI. A previous investigation found bilateral decreased volume in the parahippocampal regions during the performance of a navigational task (30). This cluster may reflect more mTBI-related dysfunction. In the second cluster, we see the caudal anterior cingulate, cuneus, entorhinal, fusiform, and temporal pole. In a previous study on trigeminal neuralgia, both the cuneus and fusiform were found to be reduced and implicated in multisensory integration and cognitive processing (31). This may align with the caudal region of the anterior cingulate cortex (ACC) being found as it is involved in the more cognitive nature of performance monitoring and updating internal cognitive and motor models (32). Moreover, a form of temporal lobe epilepsy located in the temporal pole has been tied toward facial pain and trigeminal nerve trauma (33). This further aligns with work showing activation of the trigeminal nerve system in persons with PTH [see (3) for review]. Together, findings may point toward two clusters that relate to the independent impact of the mTBI and PTH.

Proposed ML models integrating subjective self-reporting with objective brain imaging data present competitive accuracies relative to existing models. Using resting-state EEG waveforms in combination with a DL algorithm to differentiate persons with mTBI from healthy controls produced an accuracy of over 90% (34). The use of dynamic functional connectivity has been shown to yield accuracies of 92% using an SVM approach (linear kernel) (16, 17). There has been one prior investigation using ML with post-traumatic headache (13), finding that adults with persistent PTH were best classified, on average, from persons with migraine using both questionnaire and imaging data (average accuracy was 78.05%); however, this did not address clinical from non-clinical participants. Our work evaluated a PTH cohort relative to healthy controls in the pediatric and young adult age range, an important distinction in the context of pain considering ongoing neurodevelopmental processes (35, 36). Although their group used diffusion and morphometric data, preventing direct comparison with our use of only morphometric data, it is interesting to compare clinical/questionnaire data. Whereas their behavioral metrics reflected the use of a concussion survey tool, and a sub-scale of the BDI, our metrics were very pain centric. In fact, there were no pain-related scales in their analysis. Moreover, we show in our analysis the role of parental/caregiver responses, which highlights inherent limitations with pediatric self-report data. As such, our investigation extends their findings by showing the relevance of using both subjective and objective features in a pediatric and young adult PTH cohort and the specific role of pain metrics in classifying a headache cohort.

There are limitations to this investigation. (1) Population size: There was a low number of individuals included in this investigation based on limitations from the primary investigation. To help address this, multiple time points for everyone were integrated into the analyses to increase the number of sample sizes. These data integrate the resolution and persistence of PTH symptoms; however, future investigations with more inter-subject variability may help to improve classifier performance. (2) Breadth of classifiers: We included three classifiers in this investigation as examples for the performance of subjective and objective data. There are other algorithms, such as DL algorithms and sub-tools, such as modification of non-linear kernels for SVMs, that may have impacted the performance of the included algorithms. These will be explored in future work that aimed at optimizing classifiers for persons with PTH. (3) Only structural neuroimaging was used. The use of structural MRI was done based on the literature showing the involvement of features, such as cortical thickness and brain region volume, in persons with PTH (12). This was performed knowing that there are also functional features that can aid in the classification strength of each algorithm. However, the aim of this investigation was to demonstrate the relative contribution of behavioral and MRI-based features and future investigations will be more inclusive of features that could improve classification accuracy. (4) Age range: This study included participants from 12 to 22 years of age and as such included significant diversity in neurodevelopment. Focus on particular subsets of this age range may produce different results depending on the stage of development.

## Conclusion

Post-traumatic headache is a relatively common condition experienced after an mTBI with little understanding regarding its biomechanisms. This condition that is characterized through self-reporting of headache and pain symptoms is optimally defined through the use of pain-focused metrics and objective MRI-based features. This investigation underscores the role of the patient and caregiver experience when attempting to classify persons with PTH and potentially other pain-related disorders. It is critical to find ways of integrating patient-provided features into ML algorithms where it is possible to improve both ecological validity and classification accuracy.

## Data Availability Statement

The raw data supporting the conclusions of this article will be made available by the authors, without undue reservation.

## Ethics Statement

The studies involving human participants were reviewed and approved by Institutional Review Board - Boston Children's Hospital. Written informed consent to participate in this study was provided by the participants' legal guardian/next of kin.

## Author Contributions

JM, SH, and MO'B: data collection. SH and JM: manuscript preparation. DB, DZ, MO'B, and LS: manuscript review. All authors contributed to the article and approved the submitted version.

## Funding

This work was supported by a grant from NINDS (R01NS095655) to DB.

## Conflict of Interest

The authors declare that the research was conducted in the absence of any commercial or financial relationships that could be construed as a potential conflict of interest.

## Publisher's Note

All claims expressed in this article are solely those of the authors and do not necessarily represent those of their affiliated organizations, or those of the publisher, the editors and the reviewers. Any product that may be evaluated in this article, or claim that may be made by its manufacturer, is not guaranteed or endorsed by the publisher.
